# The Table of Standard Atomic Weights—An exercise in consensus

**DOI:** 10.1002/rcm.8864

**Published:** 2022-06-22

**Authors:** Tyler B. Coplen, Norman E. Holden, Tiping Ding, Harro A.J. Meijer, Jochen Vogl, Xiangkun Zhu

**Affiliations:** ^1^ U.S. Geological Survey Reston Virginia USA; ^2^ National Nuclear Data Center Brookhaven National Laboratory Upton New York USA; ^3^ Institute of Mineral Resources Chinese Academy of Geological Sciences Beijing China; ^4^ Centre for Isotope Research (CIO) University of Groningen Groningen The Netherlands; ^5^ Bundesanstalt für Materialforschung und ‐prüfung (BAM) Berlin Germany; ^6^ Institute of Geology Chinese Academy of Geological Sciences Beijing China

## Abstract

The present Table of Standard Atomic Weights (TSAW) of the elements is perhaps one of the most familiar data sets in science. Unlike most parameters in physical science whose values and uncertainties are evaluated using the “Guide to the Expression of Uncertainty in Measurement” (GUM), the majority of standard atomic‐weight values and their uncertainties are consensus values, not GUM‐evaluated values. The Commission on Isotopic Abundances and Atomic Weights of the International Union of Pure and Applied Chemistry (IUPAC) regularly evaluates the literature for new isotopic‐abundance measurements that can lead to revised standard atomic‐weight values, *A_r_* °(E) for element E.

The Commission strives to provide utmost clarity in products it disseminates, namely the TSAW and the Table of Isotopic Compositions of the Elements (TICE). In 2016, the Commission recognized that a guideline recommending the expression of uncertainty listed in parentheses following the standard atomic‐weight value, for example, *A_r_* °(Se) = 78.971(8), did not agree with the GUM, which suggests that this parenthetic notation be reserved to express standard uncertainty, not the expanded uncertainty used in the TSAW and TICE. In 2017, to eliminate this noncompliance with the GUM, a new format was adopted in which the uncertainty value is specified by the “±” symbol, for example, *A_r_°(*Se) = 78.971 ± 0.008. To clarify the definition of uncertainty, a new footnote has been added to the TSAW. This footnote emphasizes that an atomic‐weight uncertainty is a consensus (decisional) uncertainty.

Not only has the Commission shielded users of the TSAW and TICE from unreliable measurements that appear in the literature as a result of unduly small uncertainties, but the aim of IUPAC has been fulfilled by which any scientist, taking any natural sample from commerce or research, can expect the sample atomic weight to lie within *A_r_* °(E) ± its uncertainty almost all of the time.

## 1 INTRODUCTION

The traditional concept of the atomic weight of a chemical element goes back more than two centuries. It was considered a constant of nature that had a single true value, which referred to the major source of that element in nature, provided for the benefit of trade, science, and education. This value was determined by chemical means. These values were provided without any estimated indication of corresponding uncertainties in the recommended values. During the 19th century, each country provided its own recommended atomic‐weight values based on its expert measurement of these values. The values often varied from country to country, which created a problem in international trade of chemicals. At the end of the 19th century, an international committee (the International Committee on Atomic Weights) was created to provide recommended atomic‐weight values for international use. In 1919, the International Union of Pure and Applied Chemistry (IUPAC) took over this task of evaluating and disseminating standard atomic weights, *A_r_* °(E), of element E from critically assessed, published information through its Commission on Isotopic Abundances and Atomic Weights (and predecessors, referred to in the following as the Commission).

Many in the scientific public do not have a full understanding of the amount of effort and the processes that occur regularly to update standard atomic‐weight values and continuously improve the Table of Standard Atomic Weights (TSAW). For example, the following questions are pertinent:

- How are new standard atomic‐weight values determined, and what are the guidelines for updating them?
- What are the uncertainties on standard atomic‐weight values—standard uncertainties or expanded uncertainties?
- What is the new format for expressing standard atomic‐weight values and their uncertainties to make it clear that they are expanded uncertainties?
- Why do 14 elements now have standard atomic‐weight values expressed as intervals?
- When an element has a standard atomic‐weight value expressed as an interval, what single value is provided for use in education, commerce, and trade?
- Why do 34 elements have no standard atomic weight?

This article was prepared to provide answers to these and other questions about standard atomic weights and the TSAW.

### 1.1 Standard atomic weight

During the second and third quarters of the 20th century, physical determinations of atomic‐weight values, for example, mass‐spectrometric methods, became available. These methods improved atomic‐weight values and are now the basis for most new values published by IUPAC. Information on the variability of atomic‐weight values from different sources of elements began to appear in the 1950s. Uncertainty information on all elements appeared for the first time in 1970, a half century after IUPAC took responsibility for atomic‐weight values.^1^ The variability in minor sources of atomic‐weight values was initially noted via footnotes to the atomic‐weight tables. A major change in the concept of atomic‐weight values and their uncertainties occurred at a Commission meeting in 1979.^2^ The Commission adopted the following definition of atomic weight (mean relative atomic mass) of an element from a specific source: “the ratio of the average mass per atom of the element to 1/12 of the mass of an atom of ^12^C.” The novelty of this definition was emphasized by the following clarifying remarks:^2^

- Atomic weight can be defined for any sample.
- Atomic weights are evaluated for atoms in their electronic and nuclear ground states.
- The “average mass per atom” in a specified source is the total mass of the element divided by the total number of atoms of that element.
- Dated tables of Standard Atomic Weights published by the Commission refer to our best knowledge of the elements in natural terrestrial sources.

An atomic weight could be defined for any sample in nature, and thus, it was acknowledged that different sources of elements may differ in their atomic weight because of differences in isotopic composition. This new definition of atomic weight led to the modern concept, which is now called a standard atomic weight, whose value corresponds to all possible sources of that element as found in natural terrestrial samples (excluding extraterrestrial materials and commercial materials with an undisclosed or inadvertent isotopic fractionation). This changed how recommended values have been presented in the most recent 40 years compared with the previous 180 years.

This new definition had several implications. It meant that dated TSAWs published by the Commission would refer to the best knowledge of the elements in natural terrestrial samples from all measured sources and would no longer refer to the atomic‐weight value of the major source of an element found in nature and that was used in trade and commerce. It also removed natural variations in isotopic ratios from a footnote in the TSAW and incorporated these variations into the uncertainty value listed in the Table proper. Standard atomic weights would be expressed relative to the mass of an unbound carbon‐12 atom and would be dimensionless by definition.^2^ Atomic weights are dimensionless numbers numerically equal to the molar masses of the elements when expressed in grams per mole. There are numerous examples on the Internet of atomic‐weight values being expressed in atomic mass unit (amu). For example, “oxygen's atomic weight is 16.00 amu, where 1 amu = 1.661 × 10^–24^ g.” Use of amu was based on the oxygen mass scale, which should have been discontinued with the publication of the 1961 TSAW^3^ following the recalculation of standard atomic weights to the carbon‐12 scale with values expressed in the unified atomic mass unit, u.

Although “relative atomic mass” is preferred by some over “atomic weight,” there has been substantial, heated discussion on this issue, and a consensus on the meaning of atomic weight was achieved during the 1975 and 1977 IUPAC General Assemblies, as documented by De Bièvre and Peiser.^4^ IUPAC and its Inorganic Chemistry Division side with “atomic weight” because they have retained a Commission named the Commission on Isotopic Abundances and Atomic Weights.

### 1.2 Uncertainties in atomic‐weight values

The importance of knowledge of the uncertainty of a value was recognized decades ago by Churchill Eisenhart, a long‐time mathematician at the U.S. National Bureau of Standards (now the U.S. National Institute of Standards and Technology, NIST), who stated that a reported value whose accuracy is entirely unknown is worthless.^5^ At the opposite extreme, the mathematician Cassius J. Keyser^6^ has stated that absolute certainty is a privilege of uneducated minds and fanatics. It is for scientific folk an unattainable ideal.

The Commission has two equally important aims: to disseminate atomic‐weight data with the highest precision and to disseminate the most reliable atomic‐weight data, that is with the highest confidence. The Commission regularly faces the challenge of balancing between the highest precision in the TSAW (largest number of significant figures and smallest uncertainty values) and near‐perfect reliability. It is a never‐ending task to weigh the published experimental evidence element by element and, often, source by source.^7^, ^8^ Beginning with a standard uncertainty, which can be thought of as plus or minus one standard deviation, when all uncertainty contributions of a measurement are combined with the same level of confidence (that of ±1 SD), the result is a combined standard uncertainty, *u_c_.* ^9^ The combined standard uncertainty may be thought of as equivalent to “±1 SD.”^9^ The Commission wishes to express atomic‐weight values with a high level of confidence. This rescaling is performed by multiplying the combined standard uncertainty by a coverage factor, *k*, which gives a quantity that is called the expanded uncertainty, *U.* ^9^ Therefore, *U* = *ku_c_.* The confidence levels (for normal distributions) for coverage factors of 1, 2, and 3, respectively, are ~68%, ~95%, and ~99.7%.^9^ The Commission aims at disseminating value pairs of standard atomic weight and uncertainty, *A_r_* °(E) and *U*[*A_r_* °(E)], such that it can claim at a high level of confidence that any element in question in all known normal sources will have an atomic weight that will not differ from the relevant *A_r_* °(E) by more than *U*[*A*_r_°(E)]. At an even higher level of confidence, bordering on complete certainty, any chemist sampling any given “normal” material,^10^ be it any ore in trade, any product at a chemical plant, or any substance at any chemical laboratory, shall be justified in expecting all elements in that material to possess atomic weights within the implied tabulated ranges of the standard atomic‐weight values.^7^, ^8^

When he first became chairman of the International Committee on Atomic Weights (ICAW) after World War II, Edward Wichers preferred that most recommended atomic weight values should carry neither experimental uncertainties nor any indication of variability among terrestrial sources.^3^ He feared that users might lose confidence in the remarkable reliability of these values. Wichers explained the ICAW atomic‐weight values as follows: the ICAW reviews the published literature and arrives at a best current value, rounds to an abbreviated value much more likely to be closer to the true atomic weight than a value whose terminal digit differs from the abbreviated value by only 1, that is, one unit in the terminal digit.^3^

To achieve its aim of providing highly reliable and precise standard atomic‐weight values, at the 1983 Commission meeting in Lyngby, Denmark,^11^ a working party was formed to examine procedures that had been used to assign uncertainties to standard atomic‐weight values. The working party reported to the Commission at the next meeting in Lyon, France. Its recommendations and unpublished “Technical Guidelines” were adopted by the Commission.^12^ Subsequently, they were modified at the 1995 Commission meeting at Guildford, UK (see Supporting Information).^13^ One of the issues addressed by the working party was a decision adopted in the 1981 TSAW^14^ of using uncertainties of only ±1 and ±3 in the last published digit. It was recognized that the use of the single digits 1 through 9 might enable the Commission to provide more precise atomic‐weight values. This recommendation was adopted as rule 3 (see Supporting Information) and states that uncertainties be quoted (or implied) by single‐digit values (1–9) applicable with both signs to the last decimal of each tabulated standard atomic weight. As a result, the standard atomic weight of silver tabulated in the 1981 TSAW as 107.8682 ± 3 was expressed in parenthetic notation and with more precision in the 1985 TSAW as 107.8682(2). All uncertainties in a modern TSAW are single digits except for one. At the Commission meeting in 2013, the standard atomic weight of ytterbium was changed from 173.054(5) to 173.045(10) to provide a more precise standard atomic weight.^15^

### 1.3 Variability in measured isotope ratios in nature leads to asymmetric atomic‐weight uncertainties

One impact of the new definition of atomic‐weight values in 1979 was that the uncertainty in the atomic‐weight value for those elements with only two stable isotopes became asymmetric; that is, the uncertainty on the high side of the atomic‐weight value was no longer the same as that on the low side. This led to a major problem that has persisted for the past four decades. The midpoint of these symmetric uncertainties might correspond to atomic‐weight values that did not apply to any known source of that element found in terrestrial samples in nature. The natural solution to this problem would be the introduction of asymmetric uncertainties. Unfortunately, the Commission has consistently rejected that solution. Eventually, this led to the introduction of assigning atomic‐weight intervals to selected elements to avoid the problem of recommending incorrect mean values for some elements when symmetric uncertainties were retained, as discussed later.

### 1.4 The International Union of Pure and Applied Chemistry (IUPAC)

From its inception in 1919, IUPAC took over the careful evaluation and dissemination of atomic weights from critically assessed, published information through its Commission on Isotopic Abundances and Atomic Weights (and predecessors). The Commission regularly publishes a TSAW with updated standard atomic weights and a Table of Isotopic Compositions of the Elements (TICE). During the past half century, the TSAW was published biennially to publication of the 2013 TSAW^16^, the most recent TSAW. The 2021 TSAW was published in 2022.^17^ The TICE has been published at 6‐ or 8‐year intervals and was last published in 2013.^18^ The Commission has several subcommittees. Working through its Subcommittee on Isotopic Abundance Measurements (SIAM), the peer‐reviewed literature is evaluated biennially for new isotopic‐abundance measurements that might lead to revised standard atomic‐weight values. SIAM searches for new “best measurements” using mass spectrometry of isotopic abundances of an element from a single terrestrial source, preferably an isotopic reference material. The best measurement is defined “as a set of analyses of the isotope‐amount ratio or isotope‐number ratio of an element in a well‐characterized, representative material with small combined uncertainty.”^16^ New best measurement values are incorporated in the next TICE. The Commission's Subcommittee on Natural Assessment of Fundamental Understanding of Isotopes (SNAFUI) provides long‐term thinking that leads to improved clarity in tables disseminated by the Commission. For example, standard atomic weights apply only to “normal materials,” which was redefined in 2017.^10^ At approximately 20‐year intervals, the Commission publishes a review of the atomic weights of the elements that describes the gradual evolution of knowledge, understanding, and detailed information on the atomic weights of the chemical elements and their isotopic compositions in normal materials.^3^, ^7^, ^8^, ^19^, ^20^

**FIGURE 1:**
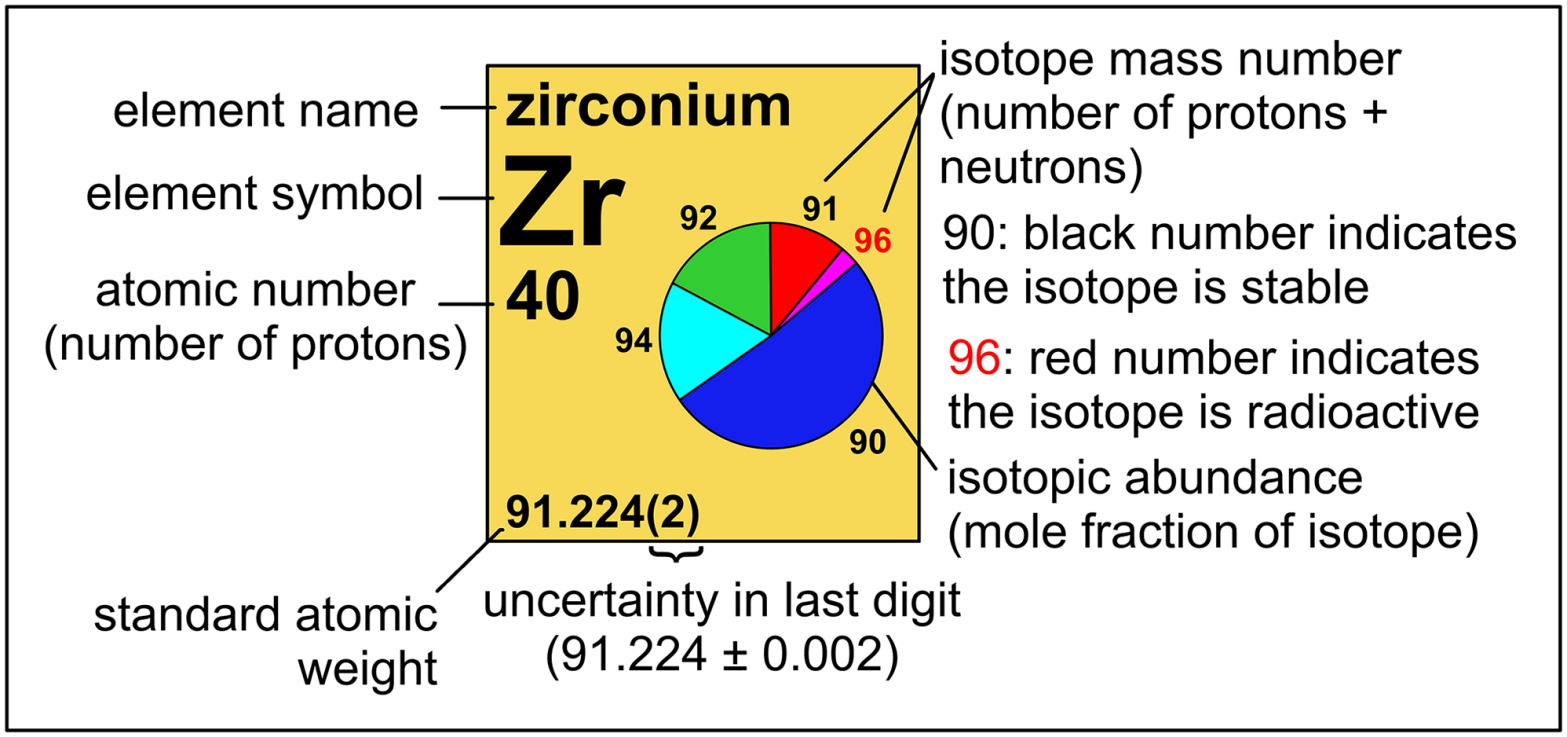
Example cell (zirconium) from the IUPAC Periodic Table of the Elements and Isotopes^21^ [Color figure can be viewed at wileyonlinelibrary.com]

A companion IUPAC high‐visibility product that is a collaboration of its Inorganic Chemistry Division and its Committee on Chemistry Education is the IUPAC Periodic Table of the Elements and Isotopes (IPTEI) for the Education Community.^21^ In addition to providing more than 400 examples of the practical use of isotopes of the elements in everyday life, a Periodic Table of the Elements and Isotopes is presented with isotopic abundances shown as pie diagrams in cells of the periodic table. Figure 1 shows zirconium. An electronic interactive version of the IPTEI, which has been designed to be used both as a stand‐alone digital learning object and as an object to be embedded in a set of electronic learning resources, can be found at www.isotopesmatter.com.

### 1.5 Normal materials

The standard atomic weights of elements apply to normal materials, and this definition was redefined in 2017 as follows:^10^

Normal materials include all substances, except (1) those subjected to substantial deliberate, undisclosed, or inadvertent artificial isotopic modification, (2) extraterrestrial materials, and (3) isotopically anomalous specimens, such as natural nuclear reactor products from Oklo (Gabon) or other unique occurrences.

This definition makes it clear that standard atomic‐weight values do not apply to extraterrestrial materials or to the decay product ^87^Sr in a natural rubidium source, but they do apply to reagents on the benchtops of chemists.

## 2 A TSAW OF THE ELEMENTS

A TSAW includes symbols, names, atomic numbers, *A_r_* °(E) values, and *U*[*A_r_* °(E)] values of elements (Table 1), and the Commission makes updates regularly. To indicate to those in the United States (and a few other countries) that “aluminium” and “caesium” are not typographic errors, the alternative names “aluminum” and “cesium” were added beginning with the 1995 TSAW.^13^ To indicate that element names are not capitalized except at the beginning of a sentence, element names were changed to lowercase beginning with the 2005 TSAW.^22^ Both these changes are a consequence of rule 8 of the Technical Guidelines (see Supporting Information), which indicates that the TSAW should be as simple, informative, and convenient to use as possible.

**TABLE 1:** Standard atomic weights

Element name	Symbol	Atomic number	Standard atomic weight		Conventional atomic weight	Foot‐notes
			Value	Uncertainty^‡^		
hydrogen	H	1	[1.007 84, 1.008 11]		1.008	m
helium	He	2	4.002 602	0.000 002		g r
lithium	Li	3	[6.938, 6.997]		6.94	m
beryllium	Be	4	9.012 1831	0.000 0005		
boron	B	5	[10.806, 10.821]		10.81	m
carbon	C	6	[12.0096, 12.0116]		12.011	
nitrogen	N	7	[14.006 43, 14.007 28]		14.007	m
oxygen	O	8	[15.999 03, 15.999 77]		15.999	m
fluorine	F	9	18.998 403 162	0.000 000 005		
neon	Ne	10	20.1797	0.0006		g m
sodium	Na	11	22.989 769 28	0.000 000 02		
magnesium	Mg	12	[24.304, 24.307]		24.305	
aluminium (aluminum)	Al	13	26.981 5384	0.000 0003		
silicon	Si	14	[28.084, 28.086]		28.085	
phosphorus	P	15	30.973 761 998	0.000 000 005		
sulfur	S	16	[32.059, 32.076]		32.06	
chlorine	Cl	17	[35.446, 35.457]		35.45	m
argon	Ar	18	[39.792, 39.963]		39.95	
potassium	K	19	39.0983	0.0001		
calcium	Ca	20	40.078	0.004		g
scandium	Sc	21	44.955 907	0.000 004		
titanium	Ti	22	47.867	0.001		
vanadium	V	23	50.9415	0.0001		
chromium	Cr	24	51.9961	0.0006		
manganese	Mn	25	54.938 043	0.000 002		
iron	Fe	26	55.845	0.002		
cobalt	Co	27	58.933 194	0.000 003		
nickel	Ni	28	58.6934	0.0004		r
copper	Cu	29	63.546	0.003		r
zinc	Zn	30	65.38	0.02		r
gallium	Ga	31	69.723	0.001		
germanium	Ge	32	72.630	0.008		
arsenic	As	33	74.921 595	0.000 006		
selenium	Se	34	78.971	0.008		r
bromine	Br	35	[79.901, 79.907]		79.904	
krypton	Kr	36	83.798	0.002		g m
rubidium	Rb	37	85.4678	0.0003		g
strontium	Sr	38	87.62	0.01		g r
yttrium	Y	39	88.905 838	0.000 002		
zirconium	Zr	40	91.224	0.002		g
niobium	Nb	41	92.906 37	0.000 01		
molybdenum	Mo	42	95.95	0.01		g
technetium*	Tc	43				
ruthenium	Ru	44	101.07	0.02		g
rhodium	Rh	45	102.905 49	0.000 02		
palladium	Pd	46	106.42	0.01		g
silver	Ag	47	107.8682	0.0002		g
cadmium	Cd	48	112.414	0.004		g
indium	In	49	114.818	0.001		
tin	Sn	50	118.710	0.007		g
antimony	Sb	51	121.760	0.001		g
tellurium	Te	52	127.60	0.03		g
iodine	I	53	126.904 47	0.000 03		
xenon	Xe	54	131.293	0.006		g m
caesium (cesium)	Cs	55	132.905 451 96	0.000 000 06		
barium	Ba	56	137.327	0.007		
lanthanum	La	57	138.905 47	0.000 07		g
cerium	Ce	58	140.116	0.001		g
praseodymium	Pr	59	140.907 66	0.000 01		
neodymium	Nd	60	144.242	0.003		g
promethium*	Pm	61				
samarium	Sm	62	150.36	0.02		g
europium	Eu	63	151.964	0.001		g
gadolinium	Gd	64	157.25	0.03		g
terbium	Tb	65	158.925 354	0.000 007		
dysprosium	Dy	66	162.500	0.001		g
holmium	Ho	67	164.930 329	0.000 005		
erbium	Er	68	167.259	0.003		g
thulium	Tm	69	168.934 219	0.000 005		
ytterbium	Yb	70	173.045	0.010		g
lutetium	Lu	71	174.9668	0.0001		g
hafnium	Hf	72	178.486	0.006		g
tantalum	Ta	73	180.947 88	0.000 02		
tungsten	W	74	183.84	0.01		
rhenium	Re	75	186.207	0.001		
osmium	Os	76	190.23	0.03		g
iridium	Ir	77	192.217	0.002		
platinum	Pt	78	195.084	0.009		
gold	Au	79	196.966 570	0.000 004		
mercury	Hg	80	200.592	0.003		
thallium	Tl	81	[204.382, 204.385]		204.38	
lead	Pb	82	[206.14, 207.94]		207.2	
bismuth*	Bi	83	208.980 40	0.000 01		
polonium*	Po	84				
astatine*	At	85				
radon*	Rn	86				
francium*	Fr	87				
radium*	Ra	88				
actinium*	Ac	89				
thorium*	Th	90	232.0377	0.0004		g
protactinium*	Pa	91	231.035 88	0.000 01		
uranium*	U	92	238.028 91	0.000 03		g m
neptunium*	Np	93				
plutonium*	Pu	94				
americium*	Am	95				
curium*	Cm	96				
berkelium*	Bk	97				
californium*	Cf	98				
einsteinium*	Es	99				
fermium*	Fm	100				
mendelevium*	Md	101				
nobelium*	No	102				
lawrencium*	Lr	103				
rutherfordium*	Rf	104				
dubnium*	Db	105				
seaborgium*	Sg	106				
bohrium*	Bh	107				
hassium*	Hs	108				
meitnerium*	Mt	109				
darmstadtium*	Ds	110				
roentgenium*	Rg	111				
copernicium*	Cn	112				
nihonium*	Nh	113				
flerovium*	Fl	114				
moscovium*	Mc	115				
livermorium*	Lv	116				
tennessine*	Ts	117				
oganesson*	Og	118				

Atomic weights are scaled to *A*_r_°(^12^C) = 12, where ^12^C is a neutral atom in its nuclear and electronic ground states, indicating that atomic weights are dimensionless numbers. Values are up to date. The atomic weights, *A*_r_(E), of many elements vary because of variations in the abundances of their isotopes in normal materials. For 14 such elements, an atomic‐weight interval is given for the standard atomic weight with the symbol [*a*, *b*] to denote the set of atomic‐weight values in normal materials; thus, *a* ≤ *A*_r_°(E) ≤ *b* for element E. For these 14 elements, single‐value conventional atomic weights for education, commerce, and industry are tabulated. The footnotes to this table elaborate the types of variation that may occur for individual elements and that may lie outside the values listed.

* Element has no stable isotopes, only radioactive isotopes. For four elements (Bi, Th, Pa, and U), a standard atomic weight is tabulated because these elements have a characteristic terrestrial isotopic composition; for the other 34 elements, a standard atomic weight cannot be determined.

^‡^ *A*_r_°(E) values and their uncertainties are given for normal materials and include evaluations of measurement uncertainty as well as natural variations in atomic weight where applicable. The atomic weight of a normal material is expected to lie within the lower and upper endpoints of the standard atomic weight with great certitude. If the uncertainty in *A*_r_°(E) is considered too large for a user's purpose for an element with measurable variations in atomic weight, a value of *A*_r_°(E) with a lower uncertainty might be obtained by measurement of an individual specimen.

^g^ Geological and biological materials are known in which the element has an isotopic composition outside the limits for normal materials. The difference between the atomic weight of the element in such materials and that given in the table may exceed the stated uncertainty.

^m^ Modified isotopic compositions may be found in commercially available material because the material has been subjected to an undisclosed or inadvertent isotopic fractionation. Substantial deviations in atomic weight of the element from that given in the table can occur.

^r^ The range in isotopic composition of the normal terrestrial material prevents a more‐precise standard atomic weight from being given; the tabulated value and uncertainty should be applicable to normal materials.

A table heading (rubric) includes relevant information that the Commission wishes to provide to users. As an outcome of efforts by the SNAFUI,^23^, ^24^, ^25^ *U*[*A*_r_(E)] values are tabulated in their own column.

The values in Table 1 are up to date,^15^, ^16^, ^26^, ^27^, ^56^ and Table 1 is based on the 2013 TSAW.^16^ A new TSAW^17^ was published in 2022 and all the values in it and Table 1 are in agreement. There are 34 elements in Table 1 with asterisks (*) that identify elements that do not have a standard atomic weight because they have only radioactive isotopes and their half‐lives are sufficiently short that they do not have a characteristic terrestrial isotopic composition. An example is radium. Because they do not have a characteristic terrestrial isotopic composition, these elements do not have an entry in the TICE.^18^ In the IPTEI, the background color of these element cells is white.^21^ Prior to the 1983 TSAW, a longest half‐life isotope, a most‐abundant isotope, another radioactive isotope, or an atomic mass number was tabulated for these 34 elements. Twenty‐one elements in Table 1 have one isotope that determines their standard atomic weight. These elements have an isotope‐amount fraction of 1 in the TICE,^18^ and the uncertainty arises entirely from the measurement of the atomic mass of the isotope. In the IPTEI, the background color of these element cells is blue.^21^ Forty‐nine elements have two or more isotopes that are used to determine their standard atomic weights. The isotopic abundances of each of the isotopes used to determine the standard atomic weight are tabulated in the TICE. In the IPTEI, the background color of these element cells is yellow.^21^ Currently, 14 elements have their standard atomic weights expressed as intervals as discussed later, and the background color of these element cells is pink.^17^, ^21^, ^54^

### 2.1 Annotations and footnotes

The importance of annotations and footnotes in the TSAW is highlighted by Peiser et al:^7^, ^8^

For quite extraordinary occurrences and other abnormal sources with abnormal atomic weights outside an otherwise acceptable range, the Commission uses annotations given in footnotes that are an integral part of the Tables of Standard Atomic Weights. Describing such abnormalities merely in the text of biennial reports would surely cause the warnings to be overlooked by more of the affected users.

Footnote “g” (Table 1) identifies elements for which geological or biological materials are known in which the isotopic composition and atomic weight are outside the limits for normal materials. Footnote “m” (Table 1) identifies elements for which modified isotopic compositions may be found in commercially available material because of undisclosed or inadvertent isotopic fractionation. Footnote “r” (Table 1) identifies elements (currently six) whose range in the isotopic composition in normal materials prevents a more‐precise standard atomic weight from being given. Footnote “*” is attached to element names and identifies the 38 elements having no stable isotopes, and 34 of these elements have no standard atomic weight because they do not have a characteristic terrestrial isotopic composition. In natural terrestrial substances, a radioactive isotope with a sufficiently long half‐life is said to have a characteristic terrestrial isotopic composition, for example, xenon‐136, potassium‐40, and protactinium‐231 (half‐life = 3.25 × 10^4^ a), and these isotopes are included in the TICE. A standard atomic weight is tabulated for bismuth, thorium, protactinium, and uranium, which have characteristic terrestrial isotopic compositions.

### 2.2 A new column and footnote for standard atomic‐weight uncertainty

About 8 years after the Commission began expressing *U*[*A*_r_°(E)] values using parenthetical notation in the 1985 TSAW^12^ (see Supporting Information), the International Organization for Standards/International Electrotechnical Commission published the first “Guide to the Expression of Uncertainty in Measurement (GUM).”^28^ In the GUM, which is now published by the Joint Committee for Guides in Metrology,^29^ the value within parentheses is the combined standard uncertainty. Thus, the Commission's expression of *U*[*A*_r_°(E)] values using parenthetical notation, such as *A_r_* °(Se) = 78.971(8), is at odds with the GUM because *U*[*A*_r_°(E)] values are not combined standard uncertainties but expanded uncertainties. To eliminate the noncompliance with the GUM, *A_r_* °(E) values and their decisional uncertainties need to be expressed in an alternative format. Alternative formats were considered (see section 2 of Supporting Information), and a format to delineate the uncertainty value with the symbol “±”, for example*, A*_r_°(Se) = 78.971 ± 0.008, was adopted.

To clarify that the tabulated uncertainties in this new column are consensus values, not GUM‐evaluated values, the column heading is identified with the symbol double dagger (‡) for a new footnote which was slightly modified by the Commission in 2021.^17^, ^25^ This new footnote in the TSAW is the first in more than two decades:^32^

‡ *A*_r_°(E) values and their uncertainties are given for normal materials and include evaluations of measurement uncertainty as well as natural variations in atomic weight where applicable. The atomic weight of a normal material is expected to lie within the lower and upper endpoints of the standard atomic weight with great certitude. If the uncertainty in *A*_r_°(E) is considered too large for a user's purpose for an element with measurable variations in atomic weight, a value of *A*_r_°(E) with a lower uncertainty might be obtained by measurement of an individual specimen.

### 2.3 Elements whose standard atomic weights are expressed as an interval

#### 2.3.1 Subcommittee on Natural Isotopic Fractionation

**FIGURE 2:**
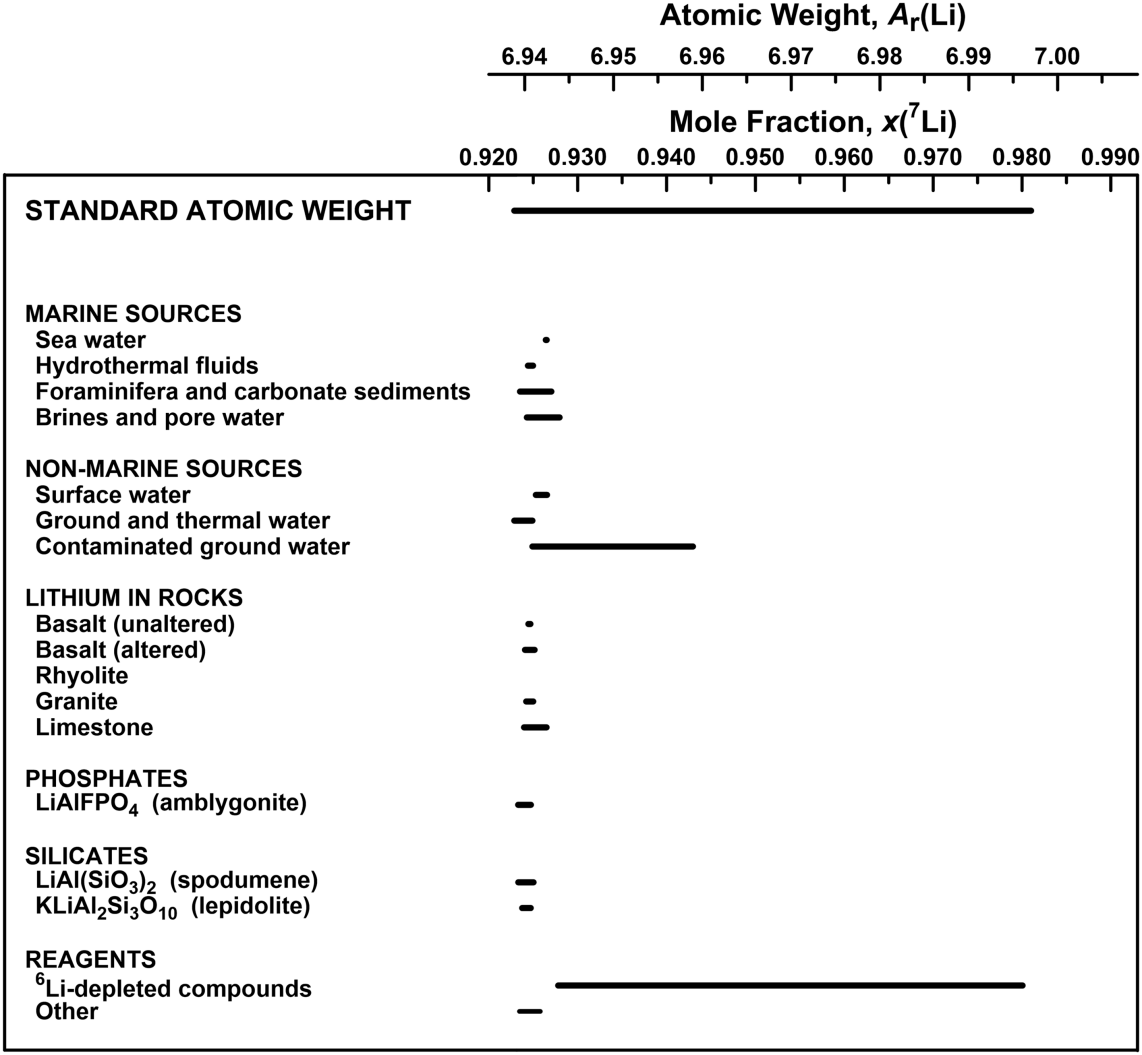
Variation in atomic weight with isotopic composition of selected lithium‐bearing materials (modified from Wieser and Coplen^30^). LSVEC is the lithium carbonate isotopic reference material for the lithium isotope‐delta scale,^38^ which is assigned an isotope‐delta value of zero.^39^ The *δ* ^7^Li_LSVEC_ isotope‐delta scale and the ^7^Li‐mole‐fraction scale were matched using the data of Qi et al.^40^ The expanded uncertainty in matching the atomic‐weight and ^7^Li‐mole‐fraction scales with the *δ* ^7^Li_LSVEC_ scale is equivalent to 3 ‰. The lower bound of the lithium standard atomic‐weight interval is 6.938, and the upper bound is 6.997. The relatively high mole fraction of ^7^Li in reagents is a result of surreptitious extraction of ^6^Li for nuclear purposes^41^ and the stealthy return of the remaining lithium to commerce for incorporation into lithium reagents^42^

**FIGURE 3:**
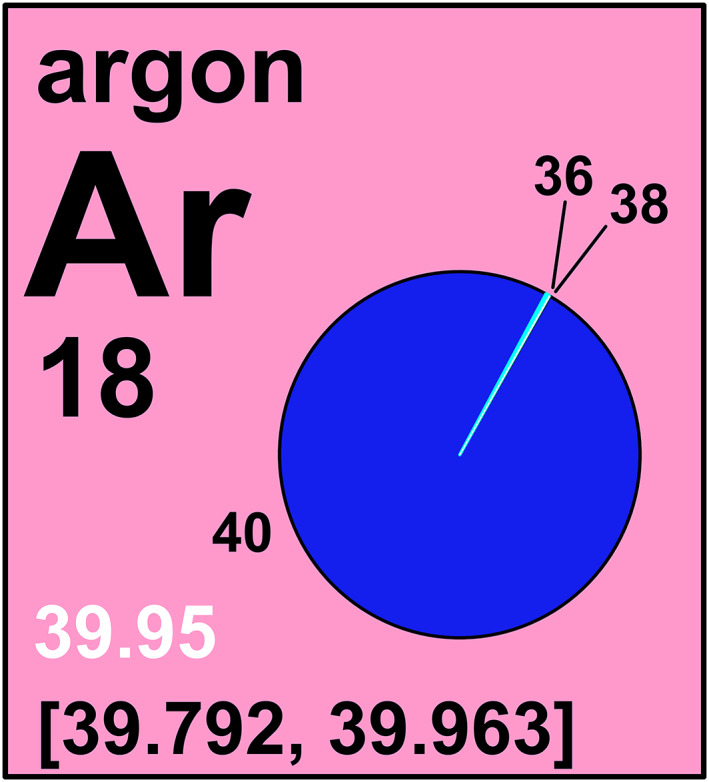
Lead element cell of the IUPAC Periodic Table of the Elements and Isotopes.^21^ The pink background exemplifies elements having two or more isotopes that are used to determine their standard atomic weights. The isotopic abundances and atomic weights vary in normal materials; these variations exceed measurement uncertainty and are well known; standard atomic‐weight values are given as lower and upper bounds within square brackets, []. The conventional atomic weights, such as for trade, commerce, and education, are shown in white [Color figure can be viewed at wileyonlinelibrary.com]

Of the 118 elements, 14 elements having standard atomic weights are expressed as intervals. During the Commission's meeting in 1985 in Lyon, France,^12^ the Working Party on Natural Isotopic Fractionation (subsequently named the Subcommittee on Natural Isotopic Fractionation) was formed to investigate the effects of isotope‐abundance variations of elements on their standard atomic weights and atomic‐weight uncertainties.^33^, ^34^, ^35^, ^36^, ^37^ That standard atomic weights vary in naturally occurring materials is shown in Figure 2, which shows the variation in atomic weight with isotopic composition of selected lithium‐bearing materials. Although the subcommittee was disbanded in 2002, its reports formed the basis of the Commission's decision in 2009 to express standard atomic weights of 10 elements (hydrogen, lithium, boron, carbon, nitrogen, oxygen, silicon, sulfur, chlorine, and thallium) as intervals, in part to indicate that standard atomic weights are not always constants of nature.^30^, ^43^ In 2011, the Commission decided to express the standard atomic weights of two additional elements (magnesium and bromine) as intervals.^31^ In 2015, the Commission expressed the standard atomic weight of argon as an interval.^27^ Most recently,^17^, ^56^ the Commission expressed the standard atomic weight of lead as an interval with *A_r_* °(Pb) = [206.14, 207.94]. Its “pie diagram” in the IPTEI^21^ is shown in Figure 3. The background color of these element cells is pink.^21^

#### 2.3.2 Conventional atomic‐weight values

In 2009, the Commission recognized that some users of atomic‐weight data of elements having interval atomic weights need only single values, such as for education, trade, and commerce. For these users, the Commission provided conventional atomic‐weight values.^16^, ^30^, ^31^ The 14 conventional atomic weights are tabulated in a separate column in Table 1. These consensus values were selected so that most or all atomic‐weight variation in normal materials is covered in an interval of ±1 in the last digit. The conventional atomic weight of lead is shown in white in Figure 3.

#### 2.3.3 Intervals and lower‐ and upper atomic‐weight bounds

The span of atomic‐weight values in normal materials is termed the “interval.” The interval [*a*, *b*] is the set of values of *x* for which *a* ≤ *x* ≤ *b*, where *b* > *a* and where *a* and *b* are the lower and upper bounds, respectively.^44^ Writing the standard atomic weight of lithium as [6.938, 6.997] indicates that its atomic weight in any normal material will be greater than or equal to 6.938 and will be less than or equal to 6.997. The atomic‐weight interval is said to encompass atomic‐weight values of all normal materials. The range of an interval is the difference between *b* and *a*, that is, *b* – *a*;^44^ thus, the range of the atomic‐weight interval of lithium is 6.997–6.938 = 0.059.

The lower bound of an atomic‐weight interval is determined from the lowest atomic weight, and it considers the uncertainty of the measurement, which is commonly an isotope‐delta measurement on a sample and a reference material, whose isotopic abundance has been measured and serves as a best measurement of a material in the TICE.^31^, ^38^ A delta measurement is a differential isotope‐ratio measurement commonly performed using an isotope‐ratio mass spectrometer. The isotope ratio of heavier and lighter isotopes ^*i*^E and ^*j*^E in a substance P and a standard std, (^*i*^E/ ^*j*^E)_P_ and (^*i*^E/ ^*j*^E)_std_,^38^ is measured. The delta value is [(^*i*^E/^*j*^E)_P_ – (^*i*^E/^*j*^E)_std_]/(^*i*^E/^*j*^E)_std_.^38^ The upper bound is determined in an equivalent manner. In addition to the uncertainty of the isotope‐delta value, the uncertainty of the isotopic reference material anchoring the isotope‐delta scale must be considered, as discussed in detail by Wieser et al.^31^ An example of the symbol for a delta value is *δ* ^7^Li_LSVEC_, shown in Figure 2, where LSVEC is the lithium isotopic reference material.^39^

The Commission's rules and comments on determining atomic‐weight intervals are listed in section 3 of the Supporting Information. Of pertinence to this discussion is rule 2:

The standard atomic weight of an element expressed as an interval, [*a*, *b*], should not be expressed as the average of *a* and *b* with an associated uncertainty equal to half of the difference between *b* and *a.* For example, *A_r_* °(C) = [12.0096, 12.0116] and should not be expressed as *A*_r_°(C) = 12.0106(48).

Exemplifying why rule 2 was enacted, Figure 2 shows a graphical plot of lithium standard atomic weight and atomic‐weight intervals for selected lithium‐bearing substances. The standard atomic weight of lithium, [6.938, 6.997], is shown at the top of Figure 2. Were one to assume that the probability distribution function of the standard atomic weight interval were rectangular (or uniform), one might express the standard atomic‐weight value as the average of the lower and upper bounds, (6.938 + 6.997)/2 = 6.9675. Figure 2 shows that this value is a poor estimate of the atomic weight of lithium. Only a laboratory reagent substantially depleted in ^6^Li could have this atomic‐weight value.

For the 14 elements whose standard atomic‐weight values are expressed as intervals, more precise atomic‐weight values of materials might be obtained by referring to published graphical plots of atomic weight for selected materials and compounds of each of these elements.^17^, ^35^, ^36^, ^37^ For example, the atomic weight of lithium in sea water is well defined as a small interval (6.942 28 ± 0.000 07).^37^ Possolo et al.^55^ provide information on determining values and uncertainties of standard atomic weight intervals of elements from specified sources.

## 3 THE PROCESS OF REVISING A STANDARD ATOMIC WEIGHT

### 3.1 Elements having one isotope to determine its standard atomic weight

**FIGURE 4:**
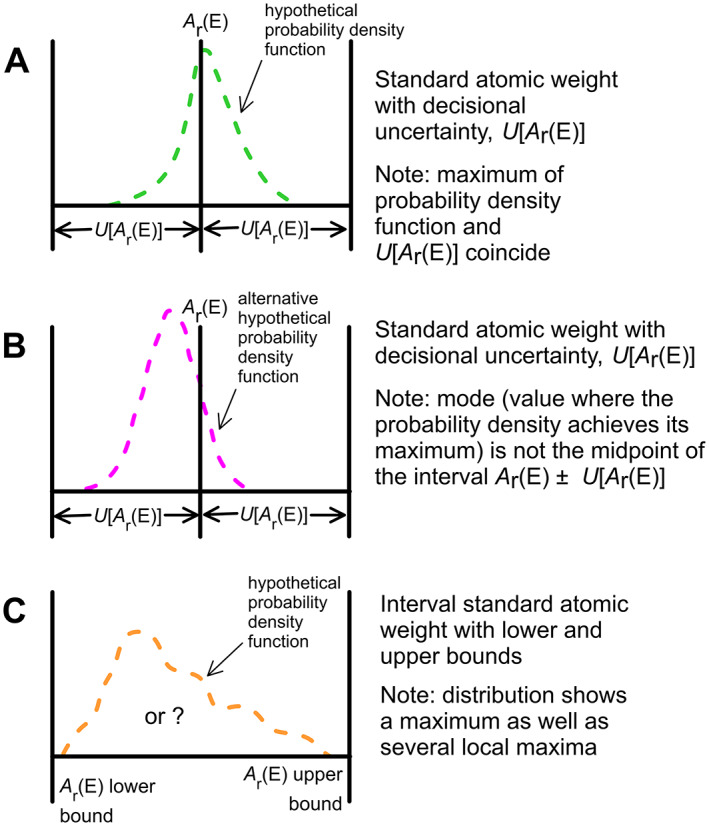
Relation among the standard atomic‐weight value, *A* *_r_* °(E) of element E, its uncertainty, *U*[*A*_r_°(E)], and the lower‐ and upper bounds of the interval standard atomic weight for various hypothetical probability density functions (modified from the element‐by‐element review^7^, ^8^). A, Example of one of the 21 elements having an *A* *_r_* °(E) value determined by a single isotope, Guide to the Expression of Uncertainty in Measurement–evaluated Gaussian uncertainty having a coverage factor of 6. B, Example of one of the 49 elements having a standard atomic‐weight value and uncertainty decided on by consensus; the highest value of the probability density function need not coincide with *A* *_r_* °(E). C, Example of one of the 14 elements having consensus standard atomic weights expressed as intervals; probability density functions are not known for these elements. Probability distribution functions are also not known for elements having footnote “r” (helium, nickel, copper, zinc, selenium, and strontium), and these may be assigned interval standard atomic‐weight values in the future [Color figure can be viewed at wileyonlinelibrary.com]

Twenty‐one elements have one isotope each to determine their standard atomic weight, which is determined from the latest Atomic Mass Evaluation.^45^ The uncertainty of each element arises entirely from the measurement of the atomic mass of this single isotope. These elements have symmetric uncertainties (expressed with the symbol ±). Since 1969,^1^ the Commission has applied a coverage factor of 6 to the GUM‐evaluated Gaussian combined standard uncertainties of atomic masses to improve the reliability of their *A_r_* °(E) values to minimize the number of changes at each revision of a TSAW (rule 10 of the Technical Guidelines in the Supporting Information). For example, the standard atomic weight of rhodium is determined from the relative atomic mass of ^103^Rh, which is 102.905 4941 ± 0.000 0025.^45^ Expanding the coverage factor by 6 yields the Commission's *A*_r_°(Rh) = 102.905 49 ± 0.000 02 value in Table 1. *A_r_* °(E)– *U*[*A*_r_°(E)] denotes the lower bound of the standard atomic weight, and *A_r_* °(E) + *U*[*A*_r_°(E)] denotes the upper bound of the standard atomic weight (Figure 4A). The *A*_r_°(E) values of these 21 elements are constants of nature.^43^ The application of rule 3 of the Technical Guidelines (see Supporting Information) to expand the uncertainty by 6 can have the result that the coverage factor will be larger than 6 owing to a reduction in significant figures. This is exemplified by the example of rhodium, which results in greater reliability of the *A*_r_°(E) values of these elements. The *A_r_* °(E) values of these elements are updated by the Commission following publication of an Atomic Mass Evaluation, which occurs at 4–10 year intervals, the most recent being published in 2017.^45^

### 3.2 Elements having two or more isotopes to determine their standard atomic weight

Forty‐nine elements have two or more isotopes to determine their standard atomic weights. These elements have symmetric uncertainties (expressed by the symbol ±). An example is hafnium with *A*_r_°(Hf) = 178.486 ± 0.006.^17^ ^,^ ^26^ The standard atomic weight is given as a single value with an uncertainty that includes measurement uncertainty and may include uncertainty due to isotope‐abundance variations. The variations in isotopic abundances may be very small and not exceed the measurement uncertainty and affect the atomic‐weight value; for example, high‐purity gallium reagents vary in isotopic composition, but the variation is too small to affect its standard atomic weight.^19^, ^46^ The following steps comprise the process to revise a standard atomic weight of these elements.

1. A researcher publishes isotopic abundances of an element with low uncertainties, commonly measured using thermal ionization mass spectrometry or multi‐collector inductively coupled plasma–mass spectrometry.
2. Beginning several months before a Commission meeting, SIAM reviews the literature, seeking peer‐reviewed publications of isotopic‐abundance measurements of samples that might achieve best‐measurement status. Preference is given to analyses of chemically stable materials that are distributed internationally as isotopic reference materials.^47^ Guidance was provided in the 2013 TSAW:^16^
  The Commission seeks evidence that mass‐spectrometer linearity, mass‐spectrometric fractionation of ions of varying masses, memory, baseline, interferences among ions, sample purity and preparation effects, and statistical assessment of data were carried out properly. Preference is given to measurements that are fully calibrated with synthetic mixtures of isotopes of the element of interest, covering the isotopic‐abundance variations of normal materials over the interval of the masses of the isotopes in the material being analyzed.
3. Meeting immediately before the biennial meeting of the Commission, SIAM conducts a full GUM‐based uncertainty evaluation on prospective best measurements.^16^, ^47^ SIAM calculates uncertainties of any new best measurement of isotopic abundances of the element, which commonly is an improvement over the existing best measurement of the element or an improvement in the documentation of factors for the uncertainty evaluation. These best‐measurement results are to appear in the next‐published TICE.
4. SIAM forwards all new best measurements of isotopic abundances of an element to the Commission.
5. The Commission, which meets after the SIAM meeting, discusses each new best measurement to determine whether it might result in a revised standard atomic weight. The Commission is aware of the rules in its Technical Booklet (see Supporting Information). For example, the aim of the Commission is to minimize the number of changes at each revision. Existing rules may be slightly compromised if the need for future changes can be reduced (rule 10). A change in a standard atomic weight and uncertainty pair is adopted only if a significant improvement in reliability or precision can be achieved (rule 9). The standard atomic weights should be given as precisely as possible, according to the guidelines adopted by the Commission, and should have as many digits as possible consistent with the other rules (rule 7).
6. For each new best measurement of an element that appears to be the basis for a revised standard atomic weight, the Commission seeks evidence for variation in isotopic composition of various sources of the element. Isotope‐delta measurements^38^ of various materials may be published, and an important link is a best measurement of isotopic abundances with low uncertainties on the isotopic reference materials used for the isotope‐delta measurements of the element—this measurement enables the isotope‐delta scale to be linked to the mole‐fraction scale.
7. The atomic weight of the best measurement is regularly used for the start of face‐to‐face discussions on a revised standard atomic weight. Typically, previous best‐measurement values and their associated revised standard atomic‐weight values have been given lesser weight during discussions, as evidenced by the paragraphs describing new standard atomic‐weight values in Commission reports during the 1980s, 1990s, and 2000s. Evidence of variation in the isotopic composition of various sources of the element requires the expansion of the uncertainty of the revised standard atomic weight. For example, the atomic weight calculated from the best‐measurement isotopic abundances of an element that shows no variation in the isotopic composition of normal materials of the element hypothetically might be 123.456 ± 0.019. Based on discussions, the Commission might then make a consensus decision that the standard atomic weight should hypothetically be 123.46 ± 0.03 so that the revised standard atomic weight encompasses the interval 123.43–123.49. This situation is exemplified in Figure 4B. The maximum of the probability density function (123.456) differs from the *A_r_* °(E) value of 123.46 (Figure 4B). The quantity *A_r_* °(E) – *U*[*A*_r_°(E)] denotes the lower bound of the standard atomic weight, and the quantity *A_r_* °(E) + *U*[*A*_r_°(E)] denotes the upper bound of the standard atomic weight (Figure 4B). If there is evidence or suspicion of variation in isotopic composition in normal materials, the Commission would probably expand the uncertainty—a hypothetical value of 123.46 ± 0.07 might be agreed on. Alternatively, with evidence of variation in isotopic abundances and source materials at 123.43 ± 0.02, the Commission might reach a consensus standard atomic weight of 123.44 ± 0.05.
8. A Commission member familiar with the measurement technique used for the best measurement commonly is assigned to write a paragraph for the next TSAW, explaining the Commission's reasoning for the revision in the standard atomic weight.
9. When *A_r_* °(E) is a consensus estimate of the true value of the atomic‐weight value of element E and *U*[*A*_r_°(E)] is a consensus‐expanded uncertainty associated with this estimate, a revised standard atomic‐weight value and uncertainty may be announced in an IUPAC news release after being approved by the Inorganic Chemistry Division of IUPAC and the IUPAC Bureau. An example is the news release for hafnium.^26^ This news release serves as a fully citable, peer‐reviewed publication.
10. The final step is publication of the revised standard atomic weight with the Commission's reasoning for the revision in the next‐published TSAW.

### 3.3 Elements whose standard atomic weights are expressed as an interval

For an element having two or more isotopes to determine its standard atomic weight to move from having a standard atomic weight with symmetric uncertainty to one having a standard atomic weight expressed as an interval, two components are required: (a) a best measurement of isotopic abundances of the element with sufficiently low uncertainties and an isotope‐delta value must both exist, and (b) a detailed investigation of peer‐reviewed, published variations in isotopic abundances in normal materials must have been completed. The literature survey is conducted by one or more experts participating in an IUPAC project.^30^ Figure 2 shows the result of a literature survey of lithium isotopic variations. Commonly, isotope‐delta measurements^38^ of various materials are published, but the missing link is a best measurement of isotopic abundances with low uncertainties on the isotopic reference materials used for the isotope‐delta measurements of the element—this measurement enables the isotope‐delta scale to be linked to the mole‐fraction scale. This link is exemplified for lithium by the text “The expanded uncertainty in matching the atomic‐weight and ^7^Li‐mole‐fraction scales with the *δ* ^7^Li_LSVEC_ scale is equivalent to 3 ‰” in the caption of Figure 2. Thirteen Commission rules and comments on determining atomic‐weight intervals can be found in section 3 of the Supporting Information.

Footnote “r” in Table 1 identifies elements whose range in the isotopic composition of normal materials prevents a more‐precise standard atomic weight being assigned, and they are good candidates for elements that may move to the category of having an atomic weight that might be expressed as an interval. These elements include helium, nickel, copper, zinc, selenium, and strontium (Table 1), and there is an ongoing project for strontium.^48^ Although probability density functions of the 14 elements having interval atomic‐weight values are not well known, the maximum values of the probability density functions should approximately align with their conventional atomic‐weight values.

## 4 PRECISION VERSUS RELIABILITY OF STANDARD ATOMIC‐WEIGHT VALUES

### 4.1 GUM uncertainty evaluations by the Commission

Although the standard atomic‐weight values and their uncertainties are consensus values, the Commission employs formal statistical data reduction tools to combine the measurement results of (a) isotopic abundance data for the determination of best measurements published in the TICE and (b) atomic‐mass data for the 21 elements having one isotope that is used to determine *A_r_* °(E) and *U*[*A*_r_°(E)] values (Figure 4A). These 21 uncertainties are GUM‐evaluated measurement uncertainties, and they are expanded with a coverage factor of 6 to achieve improved reliability.^1^ Therefore, these 21 *A*_r_°(E) values are constants of nature.

### 4.2 Decisional (consensus) standard atomic‐weight and uncertainty values

During a 2008 IUPAC project meeting,^49^ P. De Bièvre emphasized that for elements with two or more stable isotopes, the atomic‐weight value provided as a result of the Commission's consensus decision is not a measurement result, and the normal concept of uncertainty with its familiar normal or Gaussian distribution does not apply. He coined the term “decisional uncertainty,” which has been used in TSAWs since 2009.^16^, ^30^, ^31^ This concept agrees with N. N. Greenwood's argument decades earlier that the standard atomic weights are consensus values enunciated by qualified experts. The qualifier, “decisional,” appears neither in the GUM^29^ nor in the International Vocabulary in Metrology.^44^ But in the Commission's publication TICE, the best measurement of isotopic abundances of an element is a Commission outcome that has GUM‐evaluated measurement uncertainties.^16^, ^47^

For each of the 49 elements whose *A_r_* °(E) and *U*[*A*_r_°(E)] values are determined from two or more isotopes of an element (Figure 4B), the quantities *A_r_* °(E) and *U*[*A*_r_°(E)] are the Commission's decisional standard atomic‐weight value and decisional uncertainty (Figure 4B). For each of the 14 elements having a standard atomic weight expressed as an interval, each lower and upper bound is a consensus decision by the Commission based on professional evaluation and judgment.^31^ Their probability density functions (Figure 4C) are not well known, and no statistical distribution is implied. Specifically,^31^

The interval designation does not imply any statistical distribution of atomic‐weight values between the lower and upper bounds (e.g., the mean of *a* and *b* is not necessarily the most likely value). Similarly, the interval does not convey a simple statistical representation of uncertainty.

### 4.3 Reliability of atomic‐weight values

The question arises, “how reliable are the standard atomic weights of the elements?” Evidence suggests that standard atomic weights are highly reliable. In past reports, the Commission has referred to relative uncertainty, the uncertainty divided by an element's standard atomic weight, *U*[*A*_r_°(E)]/*A_r_* °(E). Between 1969 and 1997, there was no change in the relative uncertainty of 14 elements, the relative uncertainty of 69 elements improved, and only one element (xenon) had an “improvement factor” of less than 1, indicating a loss in the estimate of relative uncertainty.^50^, ^51^

A second method to measure the reliability of decisional standard atomic‐weight values and their associated decisional uncertainty values is to estimate their coverage factor. The decisional uncertainty of the standard atomic weight that the Commission determines (e.g., 0.008 for selenium) is an expanded uncertainty and is the product of a combined standard uncertainty and the coverage factor *k* or *K.* ^9^, ^29^ The coverage factor of most elements is intentionally not specified as indicated by Coplen and Peiser,^52^ who state:

Although the Commission has declined to specify the degree of expansion, *i.e.* the recommended *K* value, we believe it is expected to correspond to at least two standard deviations.

Nevertheless, the lower and upper bounds are assigned so that standard atomic weights are highly reliable and have great certitude.^7^, ^8^ For elements having two or more isotopes that contribute to the standard atomic weight, the coverage factor is sufficiently high that De Bièvre et al state:^53^

The aim of IUPAC has been fulfilled by which any chemist – taking any natural sample from research, industry, or commerce can confidently expect his or her true sample atomic weight to lie within the tabulated range with a probability far in excess of 95%.

## 5 CONCLUSIONS

IUPAC through its Commission on Isotopic Abundances and Atomic Weights, SIAM, and SNAFUI regularly improves and updates the TSAW of the elements. Through the process of estimating uncertainties from all recognized sources of error and deciding on consensus values and uncertainties of standard atomic‐weight updates, the Commission has achieved its aim of disseminating reliable, yet precise standard atomic weights. This process has minimized the publication of unreliable values. The TSAW is being continuously improved by the addition or revision of footnotes, such as the addition of a new footnote to emphasize that standard atomic‐weight values and their uncertainties are consensus values.

## Supporting information


Supporting Information S1
