# Supplementary material for: The Table of Standard Atomic Weights—An exercise in consensus
Source: Rapid Commun Mass Spectrom. 2022 Jun 22;36(15):e8864. doi: 10.1002/rcm.8864 (PMC9286417; doi:10.1002/rcm.8864)
Supplement: Supplementary file 1 — Supporting Information S1 [file RCM-36-0-s001.docx]

**Supplemental Material for “The Table of Standard Atomic Weights—an exercise in consensus”**

by

Tyler B. Coplen,^1^ Norman E. Holden,^2^ Tiping Ding,^3^ Harro A. J. Meijer,^4^ Jochen Vogl,^5^ and Xiangkun Zhu^6^

^1^U.S. Geological Survey, Reston, Virginia, USA

^2^National Nuclear Data Center, Brookhaven National Laboratory, Upton, New York, USA

^3^Institute of Mineral Resources, Chinese Academy of Geological Sciences, Beijing 100037, China

^4^Centre for Isotope Research (CIO), University of Groningen, Nijenborgh 4, 9747 AG Groningen, The Netherlands

^5^Bundesanstalt für Materialforschung und -prüfung (BAM), Berlin, Germany

^6^Institute of Geology, Chinese Academy of Geological Sciences, Beijing 100037, China

**1. Rules of the “Technical Guidelines” adopted by the Commission in 1995**

To achieve its aim of providing highly reliable and precise standard atomic-weight values, at the 1983 Commission meeting in Lyngby, Denmark, [1] a Working Party was formed to examine procedures that had been used to assign uncertainties to standard atomic-weight values. The Working Party reported to the Commission of Atomic Weights and Isotopic Abundances (CAWIA) at the next meeting in Lyon, France on those as well as on other procedures that might be used in the future, recognizing that uncertainties other than ±1 and ±3 in the last published digit were used beginning with the Commission’s 1981 TSAW. [2] These Working Party recommendations and unpublished “Technical Guidelines” were adopted by the Commission. [3] Subsequently, they were modified at the 1995 Commission meeting at Guildford, U.K., [4] and they are published here for the first time:

“The aim of CAWIA in disseminating *A*_r_(E)’s is to consider the following rules (or guidelines) more or less in declining order of importance and subject to increasing license for making compromises with other rules.

Rule 1: CAWIA recognizes that the application of the rules is subject to judgment of the Commission members and that is the intent.

Rule 2: *A*_r_(E)’s must be based on published information.

Rule 3: Uncertainties, *U* or *U*[*A*_r_(E)], must be quoted (or implied) by single digit values (1–9) applicable with both signs to the last decimal of *A*_r_(E)’s.

Rule 4: *A*_r_(E)’s must be highly reliable (defined with some vagueness) for all “normal” sources of E’s. This is also defined with vagueness, but fortunately most highly anomalous sources do not depart in *A*_r_(E) from clearly normal sources. (For a definition of “normal” refer to page 700 in S. Peiser et al., “Element by Element Review of Their Atomic Weights,” Pure Appl. Chem., 56, 695, 1984).

Rule 5: *A*_r_(E) ± *U*[*A*_r_(E)], defines the upper and lower bounds of the range of normal occurrences according to guidelines adopted by the Commission.

Rule 6: The *A*_r_(E) ± *U*[*A*_r_(E)], pair constitutes the Table entry for all elements with a characteristic terrestrial composition. The entry may be amplified by footnotes and explanations in the rubric which are an integral part of the Table.

Rule 7: The *A*_r_(E)’s should be given as precisely as possible, again according to the guidelines adopted by the Commission, and should have as many digits as possible consistent with the previously stated rules.

Rule 8: The Table should be as simple, informative, and convenient to use as possible.

Rule 9: At biennial revisions of the Table, CAWIA will recommend a change in an *A*_r_(E) ± *U*[*A*_r_(E)] pair only if a significant improvement in reliability or precision can thereby be achieved.

Rule 10: The aim of the Commission should be to minimize the number of changes at each revision and subsequent rules may be found acceptable or existing rules may be slightly compromised if, thereby, the need for changes or the probability of later changes can be reduced.

Rule 11: These rules will be applied uniformly to all elements.

Rule 12: Three months before each meeting of CAWIA, the Chairman of the Subcommittee for Isotopic Abundance Measurements (SIAM) should distribute to SIAM members all of the information on any element whose atomic weight is under consideration for change. In this way members will have sufficient time to review materials. Then, the Secretary should distribute all papers that relate to a change in the atomic weight of an element to all members of CAWIA so that each member has at least one month to review them.

Rule 13: All data under consideration by CAWIA must be subjected to analysis by the Commission’s accepted Isotopic Abundance and Atomic Weight Calculation methodology.

Rule 14: Column 4 of the Isotopic Composition Table must be reserved for verified natural isotopic fractionations, from mineral surveys and the like.”

Regarding rule 4, the definition of a normal material was updated [5] and the Commission’s calculation methodology for rule 13 was published. [6]

**2. Atomic-weight-uncertainty formats to eliminate noncompliance with the GUM**

At the request of the Commission, the Subcommittee on Natural Assessment of Fundamental Understanding of Isotopes identified three possible formats to eliminate the noncompliance:

1. Expression of the uncertainty value within square brackets, following the last significant figure to which it is attributed; *e.g.*, *A*_r_(Se) = 78.971[8].
2. Expression of the uncertainty value within curly brackets, following the last significant figure to which it is attributed; *e.g.*, *A*_r_(Se) = 78.971{8}.
3. Delineation of the uncertainty value with the symbol “±”; *e.g.*, *A*_r_(Se) = 78.971 ± 0.008.

The first possibility might be misleading because “[8]” might be misconstrued as a reference. The second choice using curly brackets is a viable possibility and a Table of Standard Atomic Weights and an Excel file using this approach is available. [7] However, the second choice was rejected by the Commission in 2017 because curly brackets may be used for other numerical quantities and ambiguity may result. The third choice was preferred and was accepted by the Commission at its meeting in 2017. It has the advantage that standard atomic weight and its uncertainty can be tabulated in separate columns (Table 1), resulting in two data columns instead of one. When values are transferred to a spreadsheet with the format “78.971(8),” it is necessary to remove “(8)” manually and copy “8” manually to the uncertainty column in the spreadsheet. An alternative is to write code to perform these data manipulations. The two-column format of [*A*_r_(E), *U*[*A*_r_(E)]] eliminates the need for these manipulations.

**3. Commission rules and comments on determining atomic-weight intervals**

The Commission’s rules and comments on determining atomic-weight intervals are listed below: [8]

1. The variation in atomic-weight values of an element is termed an atomic-weight “interval” with the symbol [*a*, *b*], where *a* and *b* are the lower and upper bounds, respectively, of the interval; thus, for element E, *a* ≤ *A*_r_(E) ≤ *b*.

2. The standard atomic weight of an element expressed as an interval, [*a*, *b*], should not be expressed as the average of *a* and *b* with an associated uncertainty equal to half of the difference between *b* and *a*. For example, *A*_r_(C) = [12.0096, 12.0116] and should not be expressed as *A*_r_(C) = 12.0106(48).

3. The atomic-weight interval encompasses atomic-weight values of all normal materials.

4. The atomic-weight interval is the standard atomic weight.

5. The atomic-weight interval and range should not be confused. The atomic-weight range is *b* – *a*, where *a* and *b* are the lower and upper bounds, respectively.

6. The lower and upper bounds commonly are determined from the lowest and highest isotope-delta values of normal materials, taking into account uncertainties of the isotope-delta measurements and uncertainty in relating the isotope-delta scale to the atomic-weight scale of an element.

7. Both lower and upper bounds are consensus values, and neither has any uncertainty associated with it.

8. The number of significant digits in the lower and upper bounds are adjusted so that uncertainty in either of the isotope-delta measurements or in the uncertainty relating the delta scale to the atomic-weight scale do not impact the lower and upper bounds.

9. The number of significant digits in the lower and upper bounds should be identical. A zero as a trailing digit in a value may be needed and is acceptable.

10. The atomic-weight interval is selected conservatively so that changes in the Table of Standard Atomic Weights are needed infrequently.

11. The atomic-weight interval is given as precisely as possible and should have as many digits as possible, consistent with the previously stated rules.

12. Values of atomic-weight intervals are updated in the Table of Standard Atomic Weights by the Commission following completion of an IUPAC project reviewing the published literature for peer-reviewed, isotopic-abundance data.

13. If the standard-atomic-weight uncertainty for an element has been expanded because of reported variation in isotopic composition in normal materials, but the Commission has not assigned an interval, a footnote “r” is retained in the Table of Standard Atomic Weights until the Commission completes an evaluation and determines lower and upper bounds from published data.

**References**

[1] Holden NE, Martin RL. Atomic weights of the elements 1983. *Pure Appl Chem.* 1984;56: 653–674. doi.org/10.1351/pac198456060653

[2] Holden NE, Martin RL. Atomic weights of the elements 1981. *Pure Appl Chem.*1983;55: 1101–1118. doi.org/10.1351/pac198355071101

[3] IUPAC. Atomic weights of the elements 1985. *Pure Appl Chem.*1986;58: 1677–1692. doi.org/10.1351/pac198658121677

[4] IUPAC. Atomic weights of the elements 1995. *Pure Appl Chem.* 1996;68: 2339–2359. doi.org/10.1351/pac199668122339

[5] Coplen TB, Holden NE, Wieser ME, Böhlke JK. Clarification of the term “normal material” used for standard atomic weights (IUPAC Technical Report). *Pure Appl Chem*. 2018;90: 1221–1224. doi.org/10.1515/pac-2017-0301

[6] Schaefer F, Taylor PDP, Valkiers S, De Bièvre P. Computational procedures for the treatment of measured or published isotope abundance data. *Int J Mass Spectrom Ion Proc*. 1994;133: 65–71. doi.org/10.1016/0168-1176(94)03930-5

[7] Coplen TB, Holden NE, Wieser ME, Böhlke JK. *Table of Standard Atomic Weights of the Elements 2015: U.S. Geological Survey Data Release*. 2017. <https://doi.org/10.5066/F7NS0SDB> (accessed December 18, 2019).

[8] Wieser ME, Holden N, Coplen TB, et al. Atomic weights of the elements 2011 (IUPAC Technical Report). *Pure Appl Chem*. 2013;85: 1047–1078. doi.org/10.1351/PAC-REP-13-03-02
